# A Web-Based Instrument for Infantile Atopic Dermatitis Identification (Electronic Version of the Modified Child Eczema Questionnaire): Development and Implementation

**DOI:** 10.2196/44614

**Published:** 2023-07-19

**Authors:** Heping Fang, Lin Chen, Juan Li, Luo Ren, Yu Yin, Danleng Chen, Huaying Yin, Enmei Liu, Yan Hu, Xiaoyan Luo

**Affiliations:** 1 Department of Respiratory Medicine, Children’s Hospital of Chongqing Medical University Chongqing Key Laboratory of Pediatrics, Ministry of Education Key Laboratory of Child Development and Disorders National Clinical Research Center for Child Health and Disorders Chongqing China; 2 Department of Child Health Care, Children’s Hospital of Chongqing Medical University Chongqing Key Laboratory of Child Health and Nutrition, Ministry of Education Key Laboratory of Child Development and Disorders National Clinical Research Center for Child Health and Disorders Chongqing China; 3 Department of Dermatology, Children’s Hospital of Chongqing Medical University Chongqing Key Laboratory of Child Infection and Immunity, Ministry of Education Key Laboratory of Child Development and Disorders National Clinical Research Center for Child Health and Disorders Chongqing China

**Keywords:** atopic dermatitis, identification, infant, web-based questionnaire, public health

## Abstract

**Background:**

Atopic dermatitis (AD) is a chronic inflammatory cutaneous disease that affects 30.48% of young children; thus, there is a need for epidemiological studies in community settings. Web-based questionnaires (WBQs) are more convenient, time-saving, and efficient than traditional surveys, but the reliability of identifying AD through WBQs and whether AD can be identified without the attendance of doctors, especially in community or similar settings, remains unknown.

**Objective:**

This study aimed to develop and validate a web-based instrument for infantile AD identification (electronic version of the modified Child Eczema Questionnaire [eCEQ]) and to clarify the possibility of conducting WBQs to identify infantile AD without the attendance of doctors in a community-representative population.

**Methods:**

This study was divided into 2 phases. Phase 1 investigated 205 children younger than 2 years to develop and validate the eCEQ by comparison with the diagnoses of dermatologists. Phase 2 recruited 1375 children younger than 2 years to implement the eCEQ and verify the obtained prevalence by comparison with the previously published prevalence.

**Results:**

In phase 1, a total of 195 questionnaires were analyzed from children with a median age of 8.8 (IQR 4.5-15.0) months. The identification values of the eCEQ according to the appropriate rules were acceptable (logic rule: sensitivity 89.2%, specificity 91.5%, positive predictive value 97.1%, and negative predictive value 72.9%; statistic rule: sensitivity 90.5%, specificity 89.4%, positive predictive value 96.4%, and negative predictive value 75%). In phase 2, a total of 837 questionnaires were analyzed from children with a median age of 8.4 (IQR 5.2-14.6) months. The prevalence of infantile AD obtained by the eCEQ (logic rule) was 31.9% (267/837), which was close to the published prevalence (30.48%). Based on the results of phase 2, only 20.2% (54/267) of the participants identified by the eCEQ had previously received a diagnosis from doctors. Additionally, among the participants who were not diagnosed by doctors but were identified by the eCEQ, only 6.1% (13/213) were actually aware of the possible presence of AD.

**Conclusions:**

Infantile AD can be identified without the attendance of doctors by using the eCEQ, which can be easily applied to community-based epidemiological studies and provide acceptable identification reliability. In addition, the eCEQ can also be applied to the field of public health to improve the health awareness of the general population.

## Introduction

Atopic dermatitis (AD) is a chronic, recurrent inflammatory cutaneous disease that affects 5% to 20% of children worldwide [1], and the prevalence of AD in children younger than 1 year is 30.48% in China [2]. Children with AD younger than 2 years are clinically defined as having “infantile AD,” which is characterized by chronic eczema, itching, and dry skin [1]. Children with infantile AD, especially severe early-onset AD, may develop food allergies, aeroallergen sensitizations, and other airway allergic diseases later in life [3-5]. Thus, the importance of infantile AD is that its management may help reduce the incidence of subsequent allergic diseases [6].

It was reported that many patients with AD may have never consulted a doctor [7], which highlights the necessity of conducting epidemiological surveys in community settings. Currently, questionnaires have become the most acceptable method [8], but most studies are carried out through traditional surveys (paper-based questionnaires or interviews), which require more human resource, materials, and time compared with web-based questionnaires (WBQs) [9-11]. Thus, it is necessary to develop a WBQ of infantile AD for more convenient, efficient, and economical epidemiological surveys.

WBQ has many advantages, such as its speed and reach, ease of use, low cost, flexibility, and automation [12], which are exactly the characteristics required for large-scale or community-based surveys. In addition, most social network platforms allow access to WBQs at present, providing great potential in the field of digital health [13]. It is not difficult to collect medical history through WBQs [14]; however, the real challenge is determining if an epidemiological study can be conducted completely through WBQs without the attendance of doctors because an exact diagnosis of the disease is required. More importantly, the results of WBQs can be different from traditional surveys in pediatrics because pediatric WBQs are essentially self-reports from a third-person perspective and may be more affected by the subjective feelings of the caregivers (especially in younger children) [15,16]. Therefore, although the existence of infantile AD can be identified by medical histories in traditional surveys [17,18], the possibility of conducting WBQs to identify infantile AD remains unknown, especially in community or similar settings.

In this paper, we first developed and validated a web-based instrument for infantile AD identification (electronic version of the modified Child Eczema Questionnaire [eCEQ]) and then implemented the eCEQ in a community-representative population to clarify the possibility of identifying infantile AD without the attendance of doctors using WBQs.

## Methods

### Study Design

**Figure 1:**
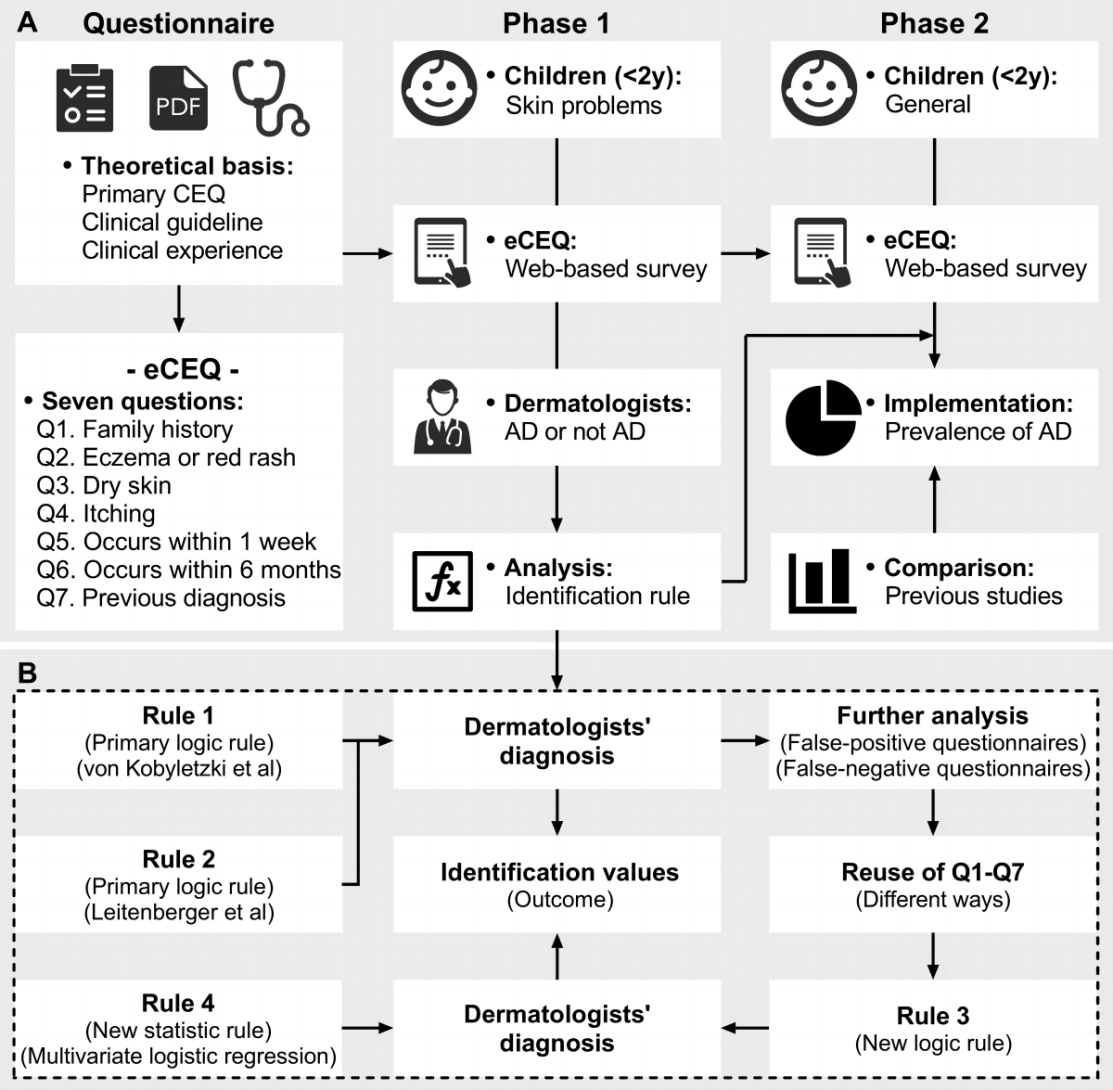
The design of this study. (A) The study protocol of phase 1 and phase 2. (B) The establishment of the eCEQ rules in phase 1 [17,18]. AD: atopic dermatitis; CEQ: Child Eczema Questionnaire; eCEQ: electronic version of the modified Child Eczema Questionnaire; WBQ: web-based questionnaire.

This study was divided into 2 phases (Figure 1A). Phase 1 developed and validated the eCEQ by comparing it with the diagnoses established by dermatologists. Phase 2 implemented the eCEQ in a community-representative population and compared the obtained prevalence with those from previous studies. The primary outcome was the reliability of the eCEQ for identifying infantile AD, whereas the secondary outcome was the possibility of identifying infantile AD without the attendance of doctors using WBQs.

### Ethics Approval

This study was approved by the Ethics Committee of the Children’s Hospital of Chongqing Medical University, China, and exempted from the signature of informed consent (2021-465). This study followed the CHERRIES (Checklist for Reporting Results of Internet E-Surveys) checklist (Multimedia Appendix 1) [19] and STROBE (Strengthening the Reporting of Observational Studies in Epidemiology) checklist (Multimedia Appendix 2) [20] to report the results.

### Study Population and Sample Size Calculations

Phase 1 was carried out in the Department of Dermatology, Children’s Hospital of Chongqing Medical University, in February 2022, and the participants were children younger 2 years who visited the department for skin problems. The sample size was determined according to the formula as described by Malhotra et al [21]. The reference sensitivity (“Sen” in the formula) and specificity (“Spec” in the formula) of the eCEQ were set as 82% and 89%, respectively (notably, these reference values correspond to rule 2 of the eCEQ in this study) [18]. The reference prevalence of infantile AD was set as 30.48% [2]. The calculation provided 187 (when calculated using the reference sensitivity) and 55 (when calculated using the reference specificity) as the minimum sample sizes when the tolerance level (“d” in the formula) was set as 10% and the confidence level was set as 95%. Thus, 187 was determined as the minimum sample size after calculation. The formulas are as follows:



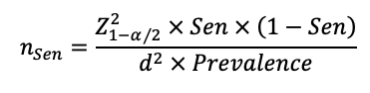





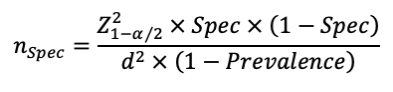



Phase 2 was carried out in the Department of Child Health Care, Children’s Hospital of Chongqing Medical University, from April to July 2022, and the participants were children younger than 2 years who visited the department for routine physical examinations. Notably, children younger than 2 years are required to receive at least six routine physical examinations in China [22], with a management rate of more than 80% [23]. Therefore, although phase 2 was conducted through convenient sampling, it still had good community representativeness. In addition, we doubled the sample size to reduce the potential effect caused by convenient sampling when calculating the sample size. The sample size was determined according to the formula summarized by Serdar et al [24]. The reference prevalence of infantile AD was set as 30.48% [2]. The calculation provided 652 as the minimum sample size when the design effect (“D” in the formula) was set as 2, the tolerance level (“d” in the formula) was set as 5%, and the confidence level was set as 95%. Furthermore, the ratio of unqualified questionnaires was expected to be 5%, and the nonresponse rate was expected to be 50% [25]. Therefore, the number of recruitments was set to be more than 1371 to obtain the sample size. The formula is as follows:



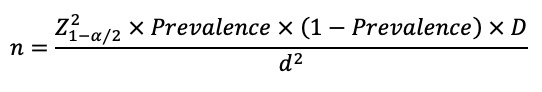



### Development and Modification of the eCEQ

The primary Child Eczema Questionnaire (CEQ) was adapted based on the questionnaire in the International Study of Asthma and Allergies in Childhood [8] to identify infantile AD through paper-based surveys, which was initially developed and validated in a Swedish population [17]. The CEQ includes 3 questions (Q1: red rash or eczema, Q2: itching, and Q3: the rash occurs in specific locations within 1 week), and the existence of AD can be identified if all the questions are answered (sensitivity: 87% and specificity: 98%; notably, this rule was defined as rule 1 of the eCEQ in this study). However, the identification values of the CEQ were not as good as described when applied to a US population (sensitivity: 72% and specificity: 93%), but it could be improved after modification (Q1: red rash, Q2: itching, and Q3: the rash occurs in specific locations within 6 months; sensitivity: 82% and specificity: 89%; notably, this rule was defined as rule 2 of the eCEQ in this study) [18], suggesting that the development of the eCEQ in Chinese populations may also need modifications.

In this study, we added several questions while developing the eCEQ based on current knowledge in case modifications are needed after analysis. First, considering the chronic, recurrent characteristics of AD, we added a question to investigate whether specific sites were involved in the last 6 months, as described by Leitenberger et al [18]. Second, we added 2 questions to the eCEQ to investigate dry skin and the family history of allergic diseases in first-degree relatives, which were important to estimate AD more comprehensively according to the Chinese guidelines for the diagnosis of AD [26]. Third, a small number of children with AD who had been diagnosed and treated could be identified as being negative for AD in a symptom-dominated questionnaire according to our experience. For this reason, we added a question to investigate the previous diagnosis of AD by a doctor. Therefore, the eCEQ included 7 questions (Table 1), and cultural adjustments were conducted by the researchers based on previous studies [27]. Additionally, to help the caregivers better self-report, we provided several pictures of typical rashes and dry skin that all participants can view automatically and easily and reminded caregivers that the pictures were only for reference.

**Table 1:** The electronic version of the modified Child Eczema Questionnaire (eCEQ).

Question	Description of the question
Q1	Whether the child’s first-degree relatives (parents and siblings) have allergic diseases (atopic dermatitis, food allergy, allergic asthma, or allergic rhinitis)
Q2	Does the child have recurrent red rashes or eczema that can come and go?
Q3	Does the child have dry skin?
Q4	Are the skin problems (rash, eczema, or dry skin) itching or scratching?
Q5	Have these skin problems (rash, eczema, or dry skin) affected the following locations in the past week: around the eyes, ears, scalp, cheeks, forehead, neck, trunk, folds of the elbows or behind the knees, wrist or ankle, or outer arms or legs?
Q6	Have these skin problems (rash, eczema, or dry skin) affected the following locations in the past 6 months: around the eyes, ears, scalp, cheeks, forehead, neck, trunk, folds of the elbows or behind the knees, wrist or ankle, or outer arms or legs? (For infants younger than 6 months, the time period is from birth to now.)
Q7	Has the child ever been diagnosed with atopic dermatitis by a doctor?

### Settings and Process of the WBQs

The questionnaires were developed through *Wenjuan.com* (a free professional WBQ platform) [28] and accessed by scanning the QR code using WeChat or other social media apps through smartphones. Considering the importance of WBQ methodology, the design, word expression, privacy protection, and data security were all based on the CHERRIES checklist (Multimedia Appendix 1) [19], the existing recommendations [12,29-31], and our current knowledge [15,32]. We collected the basic information (age and sex) of the participants through the hospital information system in phase 1, and more information was collected from the participants’ self-report in phase 2. Thus, the WBQ in phase 1 consisted of 7 questions (the eCEQ), whereas the WBQ in phase 2 consisted of 46 questions on 5 pages (notably, in addition to the basic information—such as age, sex, caregivers’ awareness of possible AD, etc—and the eCEQ, we collected additional information about family environment and health behaviors, which was part of another study and is not included in this paper). In addition, we set 1 repeated question about caregivers at the beginning and end and 1 self-evaluation question about the response quality to evaluate the reliability of the responses according to our previous studies [15,32].

The WBQs were completed anonymously and voluntarily. In phase 1, the survey was completed through self-report by caregivers in the waiting room before the visits (Figure 1A). The diagnosis of AD was made by dermatologists according to Hanifin and Rajka’s [33] criteria. The dermatologists were not allowed to know whether the patients had completed the eCEQ or not in advance. In phase 2, the recruitment of participants younger than 2 years was completed by the triage nurses by distributing recruitment materials (a small paper advertisement [Multimedia Appendix 3] and a separately packaged infant face mask as a gift), which would cover most of the children who visited the department, and duplicate participants were excluded by the nurses. The participants could read the introduction of this study and scan the QR code on advertisement to access the cover page of the WBQ (Multimedia Appendix 3). In addition, we invited 13 caregivers of children younger than 2 years to conduct a cultural adjustment before the phase 2 survey started. Additionally, we also conducted a pilot survey of 100 recruitment materials to adjust the possible unreasonableness of the process.

### Data Processing and Statistical Analysis

The collected data were directly exported as a Microsoft Excel file and double checked for potential duplicate responses (if data such as device ID, IP address, and children’s basic information were all duplicated), data reliability, age, and disease conditions (autoimmune disease, immunodeficiency disease, severe malnutrition, etc) with methods described previously [15,32].

We predetermined 4 eCEQ rules for identifying infantile AD (Figure 1B). Rules 1 and 2 were the primary logic rules that were already established [17,18]. Rule 3 was a new logic rule and was established by recombining the 7 questions based on further analysis of the false-positive and false-negative questionnaires in rules 1 and 2. Rule 4 was established through a multivariate logistic regression model, which included the 6 questions from Q1 to Q6 as the independent variables and the diagnosis by dermatologists as the dependent variable. The predictive probability was calculated by the formula summarized by Harris [34], and the optimal cut-off value of predictive probability was obtained through the receiver operating characteristic (ROC) curve. In addition, Q7 was introduced as a supplement to rule 4. The formula is as follows:



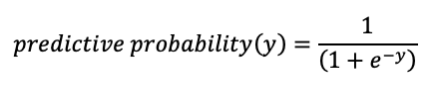



Data analysis was performed using SPSS (version 25; IBM Corp) and figures were drawn using GraphPad Prism 9 (Dotmatics) or Origin 2021 (OriginLab Corporation). Qualitative data were described as the frequencies (percentages), and quantitative data were described as the medians (IQRs) after normality testing. Chi-square and Mann-Whitney *U* tests were used to analyze the differences of age and sex between phase 1 and phase 2. To exclude the potential influence of survey setting effect in phase 2 [35], the chi-square test was used to analyze the correlation between the start times of the survey and the prevalence of infantile AD. In addition, sensitivity, specificity, positive predictive value (PPV), negative predictive value (NPV), κ coefficient, and area under the ROC curve were calculated to describe the consistency between the eCEQ rules and diagnosis of dermatologists. *P*<.05 was considered statistically significant.

## Results

### Characteristics of the Participants in Phase 1 and Phase 2

**Figure 2:**
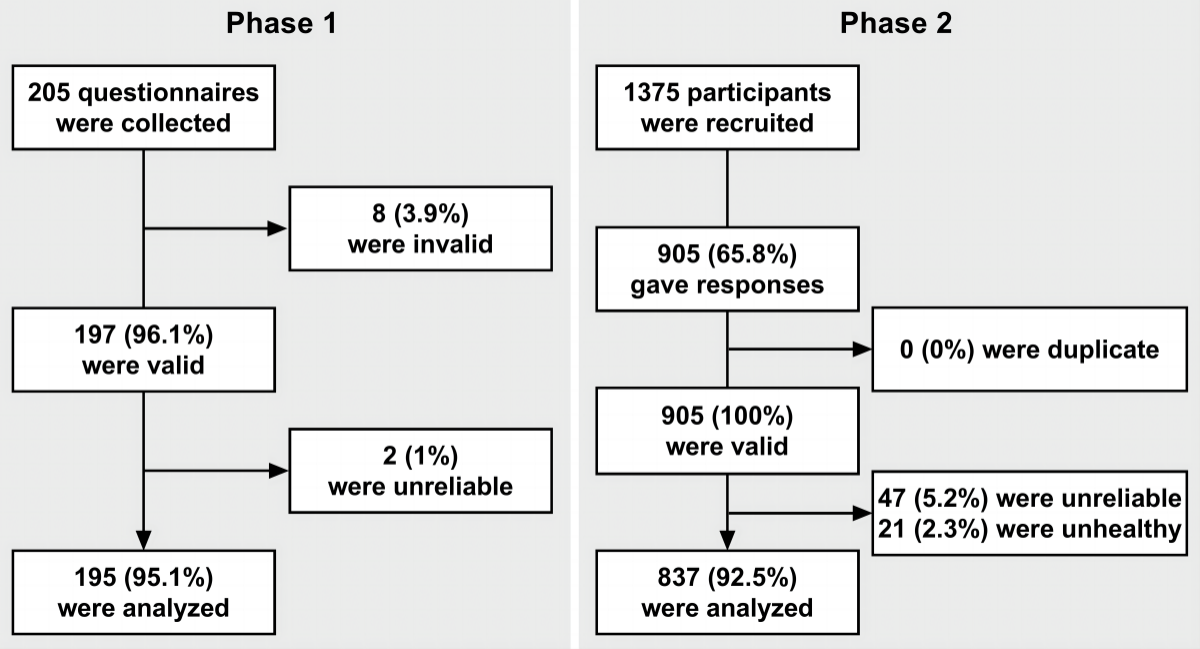
Data processing in phase 1 and phase 2.

In phase 1, a total of 205 children younger than 2 years were investigated, 195 (95.1%) of whom were included in the analysis after data processing (Figure 2). The median age of the participants was 8.8 (IQR 4.5-15.0) months, with a balanced sex ratio (99/195, 49.2% were male, and 96/195, 50.8% were female; Table 2).

In phase 2, a total of 1375 children younger than 2 years were recruited, and 905 (65.8%) participants gave responses; 837 (92.5%) of the 905 participants were included in the analysis after data processing (Figure 2). The median age of the participants was 8.4 (IQR 5.2-14.6) months, with a balanced sex ratio (439/837, 52.4% were male, and 398/837, 47.6% were female; Table 2). Subgroup analysis showed that the different start times of the survey were not correlated to the prevalence of infantile AD (*P*=.79; Figure S1 in Multimedia Appendix 4). In addition, the age (*P*=.89) and sex (*P*=.67) of the participants were not significantly different between phase 1 and phase 2.

**Table 2:** Characteristics of the participants in phase 1 and phase 2.

Characteristics			Phase 1 (n=195)		Phase 2 (n=837)
Age (months), median (IQR)			8.8 (4.5-15.0)		8.4 (5.2-14.6)
**Sex, n (%)**					
	Male	99 (49.2)		439 (52.4)	
	Female	96 (50.8)		398 (47.6)	
**eCEQ^a^, n (%)**					
	Q1. Allergic family history	116 (59.5)		255 (30.5)	
	Q2. Red rash or eczema	169 (86.7)		397 (47.7)	
	Q3. Dry skin	112 (57.4)		100 (11.9)	
	Q4. Itching	153 (78.5)		353 (42.2)	
	Q5. Occurs within 1 week	162 (83.1)		326 (38.9)	
	Q6. Occurs within 6 months	162 (83.1)		457 (54.6)	
	Q7. Previous diagnosis of AD^b^	71 (36.4)		54 (6.5)	
AD diagnosed by dermatologists in phase 1 or identified by the eCEQ in phase 2, n (%)			148 (75.9)		267 (31.9)

^a^eCEQ: electronic version of the modified Child Eczema Questionnaire.

^b^AD: atopic dermatitis.

### Development and Validation of the eCEQ Rules

**Figure 3:**
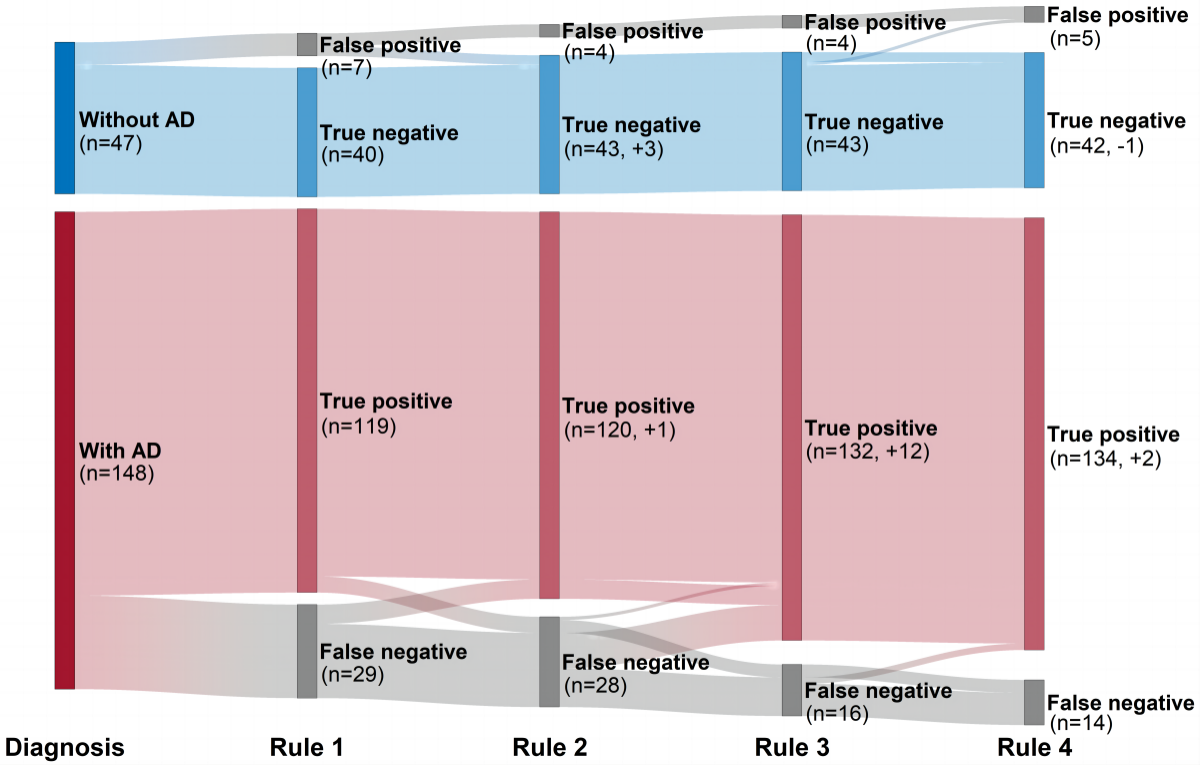
Comparison between doctor's diagnosis and the electronic version of the modified Child Eczema Questionnaire (eCEQ) rules in phase 1. AD: atopic dermatitis.

Table 3 shows the different eCEQ rules and the identification values in phase 1 (the identification values of separate questions from Q1 to Q7 are summarized in Table S1 in Multimedia Appendix 4). Although rule 2 showed better identification values than rule 1, both of them showed unsatisfactory NPVs of 58% and 60.6%, respectively. Further analysis focused on the NPV of rule 2 showed that it obtained 28 false-negative questionnaires, in which Q4 (12/28, 43%) and Q6 (9/28, 32%) were the main causes. Then, we established rule 3 based on rule 2 by introducing Q1, Q3, and Q7 as supplements to the rule in different ways. When Q1 and Q3 were introduced as supplements to Q4 and when Q7 was introduced as a supplement to the whole rule, rule 3 reduced 12 false-negative questionnaires without obtaining additional false-positive questionnaires (Figure 3).

To establish rule 4, the multivariate logistic regression model (Table 4) and ROC curve provided 0.849 as the optimal cut-off value of predictive probability. The identification values of rule 4 were more acceptable compared with rules 1 and 2 and showed similar identification values with rule 3. The κ coefficient between rule 3 and rule 4 was 0.963, and both rules were acceptable for the eCEQ to identify infantile AD.

**Table 3:** The rules of the electronic version of the modified Child Eczema Questionnaire (eCEQ) and their identification values.

Rules	Description^a^	Sensitivity (%)	Specificity (%)	PPV^b^ (%)	NPV^c^ (%)	κ	AUC^d^
1	Q2&Q4&Q5	80.4	85.1	94.4	58.0	0.57	0.83
2	Q2&Q4&Q6	81.1	91.5	96.8	60.6	0.62	0.86
3	(Q2&(Q4|(Q1&Q3))&Q6)|Q7	89.2	91.5	97.1	72.9	0.74	0.90
4	(Predictive probability>0.849)|Q7	90.5	89.4	96.4	75.0	0.75	0.90

^a^“Qn” in the formula means the answer that the participants respond to question n is “Yes.”

^b^PPV: positive predictive value.

^c^NPV: negative predictive value.

^d^AUC: area under the receiver operating characteristic curve.

**Table 4:** Multivariate logistic regression model of rule 4^a,b^.

Variables	B	β	Wald	OR^c^ (95%CI)	*P* value
Q1. Allergic family history	1.48	0.61	5.90	4.41 (1.33-14.60)	.02
Q2. Red rash or eczema	2.42	0.71	11.61	11.18 (2.79-44.86)	.001
Q3. Dry skin	1.37	0.52	6.91	3.92 (1.42-10.87)	.009
Q4. Itching	2.75	0.54	26.14	15.63 (5.45-44.83)	<.001
Q6. Occurs within 6 months	1.45	0.59	6.13	4.25 (1.35-13.39)	.01
Constant	–4.84	0.91	28.18	0.01	<.001

^a^Q5 (occurs within 1 week) was excluded because its negative predictive value for identifying infantile AD was lower than that of Q6.

^b^Logistic regression equation: y = 1.484Q1 + 2.415Q2 + 1.367Q3 + 2.749Q4 + 1.448Q6 – 4.483. Qn=0 when the answer of question n is “No”; Qn=1 when the answer is “Yes.”

^c^OR: odds ratio.

### The Prevalence of Infantile AD Identified by the eCEQ

According to rule 3 and rule 4, out of 837 participants, 267 (31.9%) and 278 (33.2%) were identified with infantile AD in phase 2, respectively. The disagreement between rule 3 and rule 4 was that 11 participants were identified with infantile AD by rule 4 but not by rule 3. Although the definite diagnosis of these 11 participants was not clear, 267 participants (rule 3) could still be identified without disagreement.

**Figure 4:**
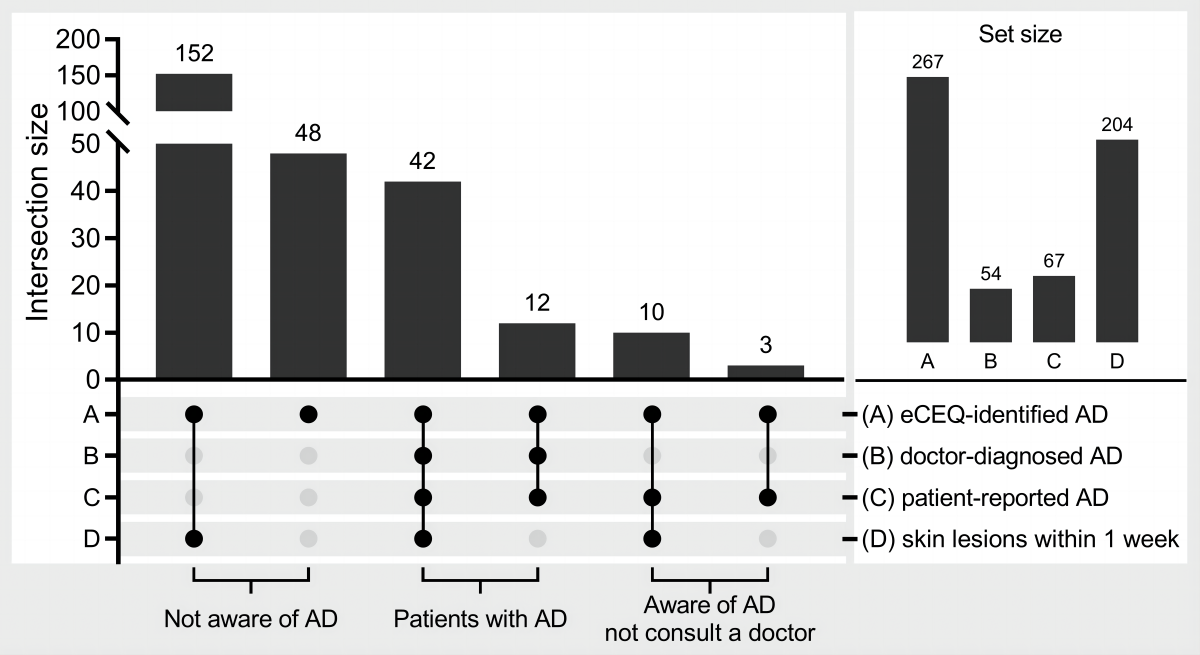
The unawareness of infantile atopic dermatitis (AD). eCEQ: electronic version of the modified Child Eczema Questionnaire.

However, only 54 (20.2%) of the 267 participants who were identified with infantile AD had been previously diagnosed by doctors. Further analysis showed that only 6.1% (13/213) of the participants who were not diagnosed by doctors but were identified by the eCEQ had been aware of the possible existence of AD. In addition, 76% (152/200) of the participants who were not aware of AD but were identified by the eCEQ had reported skin lesions within 1 week (Figure 4).

## Discussion

### Principal Findings

In this study, we developed and validated the eCEQ and then implemented it to identify infantile AD in a community-representative population. The results showed that the identification values of the eCEQ according to the appropriate rules were acceptable after being compared with the diagnosis of dermatologists, and the prevalence of infantile AD obtained by the eCEQ was close to that previously published, demonstrating the potential of the eCEQ to identify infantile AD in community-based epidemiological studies.

Epidemiological studies of AD through questionnaires are an attractive option, but the diagnosis of AD could be unreliable without doctors, which limits the value of questionnaires in such studies. In this regard, some studies applied simple questions or previous diagnoses as alternative ways to identify AD [36-38], whereas others developed survey instruments for identification [17,18,39,40]. However, although these instruments had already been validated, none of them were primarily designed as electronic versions. To the best of our knowledge, the eCEQ is the first electronic survey instrument specifically designed and validated for the identification of infantile AD in epidemiological studies. The results showed acceptable identification values in both rule 3 and rule 4. Although rule 4 was established based on multivariate logistical regression, which may make more coordinated use of each question, rule 3 seemed to be more determined and generalized because it was simpler, which can be used more conveniently in applied settings.

The eCEQ has the advantages of WBQs, but it also has several shortcomings [12,41]. First, the eCEQ is essentially a 1-way automatic medical history collection instrument. The reliability is greatly affected by the behavior and psychology of the participants when responding [42]. Second, given the differences between WBQs and traditional surveys [15,16], it may be unreasonable to expect the eCEQ to obtain the same results as traditional surveys by directly transforming the primary CEQ into an electronic version without validation. Indeed, although it could not be clarified whether the differences came from the different survey forms or cultures, or even other factors, our results showed that the identification values were not as satisfactory when applying the primary rule of the CEQ (sensitivity: 87%, specificity: 98%, PPV: 90%, and NPV: 98% [17]) to the eCEQ (sensitivity: 80.4%, specificity: 85.1%, PPV: 94.4%, and NPV: 58% in rule 1). Therefore, the existing knowledge and our results suggest that it may be necessary to treat the WBQ as a new survey method that needs validation even though it is transformed from traditional questionnaires, thus not aiming to be consistent with the traditional questionnaires but rather the real-world conditions. Taken together, it is beneficial to apply the eCEQ in epidemiological studies, but one must still consider its shortcomings when interpreting the results.

The prevalence of infantile AD identified by the eCEQ (rule 3) was 31.9%, which was reasonable for the median age of 8.4 months. Guo et al [2] investigated 5967 infants (mean age 6.24 months) through face-to-face interviews and obtained a prevalence of 30.48% for infantile AD from 12 cities in China, which is almost consistent with our results. The reported prevalence of AD in children aged 1-2 years was 30% in Chongqing and 38.71% in Chengdu [43]; both cities and their surrounding areas are the main source areas of our participants. In addition, a similar prevalence was also reported in Taiwan (infants aged 6 months) and Iceland (children aged 2 years)—33.9% and 31%, respectively [44,45]. However, although these studies demonstrated the reliability of the prevalence obtained by the eCEQ, it could not be completely ruled out that measurement errors in phase 1 and phase 2 may offset each other to produce a similar prevalence. In other words, the primary impact of this study is that it not only develops the first web-based instrument for infantile AD identification but also shows the possibility that infantile AD can be identified without the attendance of doctors using WBQs, making large-scale or community-based epidemiological research more convenient and economical.

Although the prevalence of AD was high, many patients still had not been diagnosed clinically. Saeki et al [7] reported that 36% of schoolchildren with AD had not consulted doctors. Our results showed the same problem, which was even worse in that only 20.2% of the participants identified by the eCEQ had previously been diagnosed by doctors. Further analysis showed that the possible reason was that caregivers were not aware that the skin lesions may be related to AD (only 13/213, 6.1% reported this awareness). Although health education could improve the treatment of children with AD and promote disease control [46], it is still insufficient for the general population, which could delay the diagnosis and treatment of AD. Indeed, 76% of the participants without awareness of AD but identified by the eCEQ had reported skin lesions within 1 week. In this regard, it is possible to improve the health awareness of the general population by setting automatic calculation and feedback at the end of the questionnaire or transferring the eCEQ into a self-testing app, which is another impact of this study in the field of public health.

### Strengths and Limitations

The strengths and limitations of this study are worth mentioning. First, this study developed and validated the first web-based instrument for infantile AD identification (eCEQ), which could be easily applied to epidemiological research, making large-scale or community-based surveys more convenient and economical. Second, this study focused on the possibility of identifying infantile AD without the attendance of doctors using WBQs, which is an innovative attempt and has important reference value for similar studies in the future. Third, this study also provided insights into the importance and feasible methods of public health education on infantile AD; it is important as simple instruments are more likely to serve the general public successfully.

Despite its strengths, this study has some limitations. First, the validity and reliability of the eCEQ could be overstated, as we did not recruit another sample or conduct longitudinal studies from the Department of Dermatology to further validate it in phase 1. However, the accuracy of rule 1 in the eCEQ in identifying infant AD was not substantially different from that of the primary CEQ, which still guaranteed the value of the eCEQ, especially in cross-sectional surveys. Second, this study was an anonymous cross-sectional survey; thus, the disagreement between rule 3 and rule 4 in phase 2 cannot be further clarified. Third, the eCEQ depends on the experiences of the caregivers and cannot make complex differential diagnoses, which may lead to a false-positive identification. Finally, the eCEQ was established based on a Chinese population; additional validations may be needed when it is applied to populations from different cultures and regions.

### Conclusions

In summary, we developed and validated a web-based instrument named eCEQ to identify infantile AD that can be easily used and showed acceptable reliability. We provided evidence that infantile AD can be identified without the attendance of doctors in community-based epidemiological studies. Moreover, the eCEQ can be applied to the field of public health to improve the health awareness of the general population in a convenient and economical way.

## Appendix

Multimedia Appendix 1 CHERRIES (Checklist for Reporting Results of Internet E-Surveys) checklist.

Multimedia Appendix 2 STROBE (Strengthening the Reporting of Observational Studies in Epidemiology) checklist.

Multimedia Appendix 3 The paper advertisement and cover page of the web-based questionnaire in phase 2.

Multimedia Appendix 4 Comparison between the start times of the web-based questionnaire and the prevalence of infantile atopic dermatitis (AD) in phase 2 and the identification values of separate questions from Q1 to Q7 in the electronic version of the modified Child Eczema Questionnaire (eCEQ).

## Abbreviations

AD: atopic dermatitis
CEQ: Child Eczema Questionnaire
CHERRIES: Checklist for Reporting Results of Internet E-Surveys
eCEQ: electronic version of the modified Child Eczema Questionnaire
NPV: negative predictive value
PPV: positive predictive value
ROC: receiver operating characteristic
STROBE: Strengthening the Reporting of Observational Studies in Epidemiology
WBQ: web-based questionnaire
