# Supplementary material for: A Web-Based Instrument for Infantile Atopic Dermatitis Identification (Electronic Version of the Modified Child Eczema Questionnaire): Development and Implementation
Source: J Med Internet Res. 2023 Jul 19;25:e44614. doi: 10.2196/44614 (PMC10398555; doi:10.2196/44614)
Supplement: Multimedia Appendix 1 [file jmir_v25i1e44614_app1.pdf]

Checklist for Reporting Results of Internet E-Surveys (CHERRIES)

A Web-Based Instrument for Infantile Atopic Dermatitis Identification (Electronic Version of the Modified Child Eczema Questionnaire): Development and Implementation

Item Category	Checklist Item	Explanation
Design	Describe survey design	Describe target population, sample frame. Is the sample a convenience sample? (In "open" surveys this is most likely.)<br>***Yes, in the "Study design and ethical considerations, Study population and sample size calculations" parts.***
IRB	IRB approval	Mention whether the study has been approved by an IRB.<br>***Yes, this study was approved by the Ethics Committee of the Children's Hospital of Chongqing Medical University (NO.2021-465).***
	Informed consent	Describe the informed consent process. Where were the participants told the length of time of the survey, which data were stored and where and for how long, who the investigator was, and the purpose of the study?<br>***Yes, the basic information of the questionnaire was provided, but the informed consent signature was exempted.***
	Data protection	If any personal information was collected or stored, describe what mechanisms were used to protect unauthorized access.<br>***Yes, the questionnaire was anonymous, which could not be retrieved directly by search engine and was not provided to the public template library. And the data stored in the cloud server was deleted after downloading★.***
Development and pre-testing	Development and testing	State how the survey was developed, including whether the usability and technical functionality of the electronic questionnaire had been tested before fielding the questionnaire.<br>***Yes, in the "Development and modification of the eCEQ, Settings and process of the WBQ surveys" parts.***
Recruitment process and description of the sample having access to the questionnaire	Open survey versus closed survey	An "open survey" is a survey open for each visitor of a site, while a closed survey is only open to a sample which the investigator knows (password-protected survey).<br>***No, it was a mixed-model survey.***
	Contact mode	Indicate whether or not the initial contact with the potential participants was made on the Internet. (Investigators may also send out questionnaires by mail and allow for Web-based data entry.)<br>***Yes, it was on social media mainly through WeChat app.***
	Advertising the survey	How/where was the survey announced or advertised? Some examples are offline media (newspapers), or online (mailing lists – If yes, which ones?) or banner ads (Where were these banner ads posted and what did they look like?). It is important to know the wording of the announcement as it will heavily influence who chooses to participate. Ideally the survey announcement should be published as an appendix.<br>***Yes, the recruitment of participant was completed by the triage nurses by distributing recruitment materials.***
Survey administration	Web/E-mail	State the type of e-survey (eg, one posted on a Web site, or one sent out through e-mail). If it is an e-mail survey, were the responses entered manually into a database, or was there an automatic method for capturing responses?<br>***Yes, we used web-based questionnaire based on smartphone.***
	Context	Describe the Web site (for mailing list/newsgroup) in which the survey was posted. What is the Web site about, who is visiting it, what are visitors normally looking for? Discuss to what degree the content of the Web site could pre-select the sample or influence the results. For example, a survey about vaccination on a anti-immunization Web site will have different results from a Web survey conducted on a government Web site<br>***No, not applicable.***

	Mandatory/voluntary	Was it a mandatory survey to be filled in by every visitor who wanted to enter the Web site, or was it a voluntary survey?<br>***Yes, it was voluntary.***
	Incentives	Were any incentives offered (eg, monetary, prizes, or non-monetary incentives such as an offer to provide the survey results)?<br>***Yes, a little gift was used as a response inducement measure for the survey.***
	Time/Date	In what timeframe were the data collected?<br>***Phase I was carried out in February 2022, Phase II was carried out from April to July 2022.***
	Randomization of items or questionnaires	To prevent biases items can be randomized or alternated.<br>***No.***
	Adaptive questioning	Use adaptive questioning (certain items, or only conditionally displayed based on responses to other items) to reduce number and complexity of the questions.<br>***Yes, we developed a structured questionnaire.***
	Number of Items	What was the number of questionnaire items per page? The number of items is an important factor for the completion rate.<br>***Yes, eCEQ consisted of 7 questions, while the whole questionnaire in Phase Two consisted of 46 questions. (were part of another study and would not be included in this paper)***
	Number of screens (pages)	Over how many pages was the questionnaire distributed? The number of items is an important factor for the completion rate.<br>***Yes, it was 5 pages.***
	Completeness check	It is technically possible to do consistency or completeness checks before the questionnaire is submitted. Was this done, and if "yes", how (usually JAVAScript)? An alternative is to check for completeness after the questionnaire has been submitted (and highlight mandatory items). If this has been done, it should be reported. All items should provide a non-response option such as "not applicable" or "rather not say", and selection of one response option should be enforced.<br>***Yes, the questions were required to be answered before submitting.***
	Review step	State whether respondents were able to review and change their answers (eg, through a Back button or a Review step which displays a summary of the responses and asks the respondents if they are correct).<br>***No, the responses submitted could not be reviewed or changed.***
Response rates	Unique site visitor	If you provide view rates or participation rates, you need to define how you determined a unique visitor. There are different techniques available, based on IP addresses or cookies or both.<br>***Yes, the duplicate participants were excluded by the triage nurses.***
	View rate	Requires counting unique visitors to the first page of the survey, divided by the number of unique site visitors (not page views!). It is not unusual to have view rates of less than 0.1 % if the survey is voluntary.<br>***No, not applicable.***
	Participation rate	Count the unique number of people who filled in the first survey page (or agreed to participate, for example by checking a checkbox), divided by visitors who visit the first page of the survey (or the informed consents page, if present). This can also be called "recruitment" rate.<br>***No, not applicable because we did not record the first survey page visitors.***
	Completion rate	The number of people submitting the last questionnaire page, divided by the number of people who agreed to participate (or submitted the first survey page). This is only relevant if there is a separate "informed consent" page or if the survey goes over several pages. This is a measure for attrition. Note that "completion" can involve leaving questionnaire items blank. This is not

		a measure for how completely questionnaires were filled in. (If you need a measure for this, use the word "completeness rate".)<br>***No, not applicable, but we used response rate (ratio of unique responses/unique participants) instead.***
Preventing multiple entries from the same individual	Cookies used	Indicate whether cookies were used to assign a unique user identifier to each client computer. If so, mention the page on which the cookie was set and read, and how long the cookie was valid. Were duplicate entries avoided by preventing users access to the survey twice; or were duplicate database entries having the same user ID eliminated before analysis? In the latter case, which entries were kept for analysis (eg, the first entry or the most recent)?<br>***No, we used device ID, sex, date of birth, etc. instead.***
	IP check	Indicate whether the IP address of the client computer was used to identify potential duplicate entries from the same user. If so, mention the period of time for which no two entries from the same IP address were allowed (eg, 24 hours). Were duplicate entries avoided by preventing users with the same IP address access to the survey twice; or were duplicate database entries having the same IP address within a given period of time eliminated before analysis? If the latter, which entries were kept for analysis (eg, the first entry or the most recent)?<br>***No, because different participants in the same location could have the same IP address.***
	Log file analysis	Indicate whether other techniques to analyze the log file for identification of multiple entries were used. If so, please describe.<br>***No.***
	Registration	In "closed" (non-open) surveys, users need to login first and it is easier to prevent duplicate entries from the same user. Describe how this was done. For example, was the survey never displayed a second time once the user had filled it in, or was the username stored together with the survey results and later eliminated? If the latter, which entries were kept for analysis (eg, the first entry or the most recent)?<br>***No.***
Analysis	Handling of incomplete questionnaires	Were only completed questionnaires analyzed? Were questionnaires which terminated early (where, for example, users did not go through all questionnaire pages) also analyzed?<br>***No, only completed questionnaires were analyzed, because the incomplete questionnaires were not recorded.***
	Questionnaires submitted with an atypical timestamp	Some investigators may measure the time people needed to fill in a questionnaire and exclude questionnaires that were submitted too soon. Specify the timeframe that was used as a cut-off point, and describe how this point was determined.<br>***No, because the time spent in the structured questionnaire can be different.***
	Statistical correction	Indicate whether any methods such as weighting of items or propensity scores have been used to adjust for the non-representative sample; if so, please describe the methods.<br>***Yes, the participants who were not our targets were excluded.***
