# Supplementary material for: A Web-Based Instrument for Infantile Atopic Dermatitis Identification (Electronic Version of the Modified Child Eczema Questionnaire): Development and Implementation
Source: J Med Internet Res. 2023 Jul 19;25:e44614. doi: 10.2196/44614 (PMC10398555; doi:10.2196/44614)
Supplement: Multimedia Appendix 3 [file jmir_v25i1e44614_app3.pdf]

邀请涵

“儿童皮肤健康问卷调查”

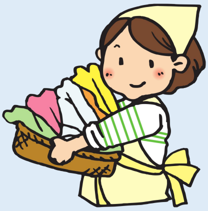

尊敬的家长朋友：

1. 我们很高兴您能够参与这项调查，这是关于“儿童皮肤健康与生活卫生行为”的匿名调查，由重庆医科大学附属儿童医院呼吸科、儿保科、皮肤科联合发起。本调查不涉及任何隐私信息，更不会侵犯您的权益。
2. 本调查大约占用您5分钟时间，您完全可以在候诊时完成填写。由于问卷是匿名的，我们希望您不要重复作答。
3. 感谢您的参与，也感谢您对儿科研究的支持，您的参与将有助于我们更好地关注儿童卫生健康问题。

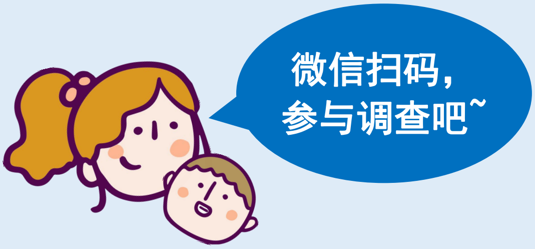

本项目获〈重庆医科大学附属儿童医院医学研究伦理委员会〉批准

研究团队联系方式：方訸平（Heping.F@outlook.com）

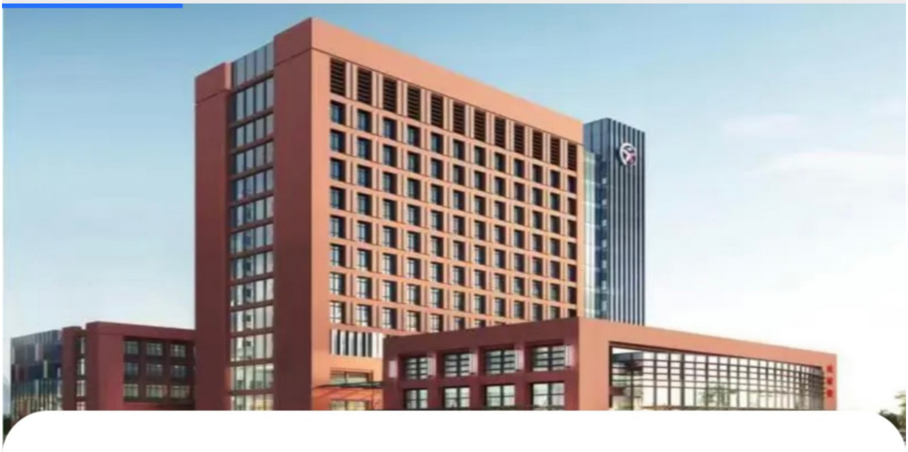

这是一项关于 **儿童皮肤健康** 与 **生活卫生行为** 的匿名调查，由重庆医科大学附属儿童医院呼吸科、儿童保健科、皮肤科共同发起。这项调查经过伦理委员会批准，**不涉及您的个人隐私信息**。

这项调查大约会占用您 **5分钟时间**，由于问卷是匿名性质的，我们希望您不要重复作答。

感谢您的参与，您的参与将有助于我们更好地关注婴幼儿的卫生健康问题。

下一页
