# Supplementary material for: A Web-Based Instrument for Infantile Atopic Dermatitis Identification (Electronic Version of the Modified Child Eczema Questionnaire): Development and Implementation
Source: J Med Internet Res. 2023 Jul 19;25:e44614. doi: 10.2196/44614 (PMC10398555; doi:10.2196/44614)
Supplement: Multimedia Appendix 4 [file jmir_v25i1e44614_app4.pdf]

A Web-Based Instrument for Infantile Atopic Dermatitis Identification (Electronic Version of the Modified Child Eczema Questionnaire): Development and Implementation

Multimedia Appendix 4:

Figure S1. Comparison between the start time of the WBQ and the prevalence of infantile AD in Phase 2. WBQ: web-based questionnaire.

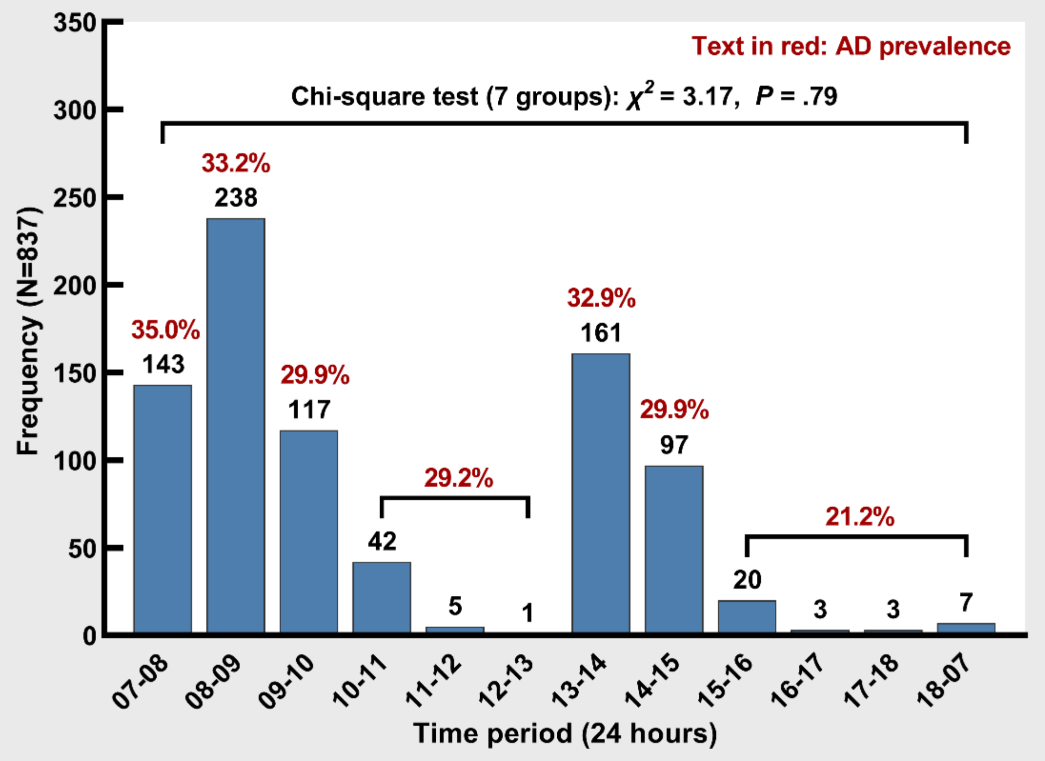

Table S1. The identification values of separate questions from Q1 to Q7 in eCEQ[a].

Questions of eCEQ	Sen[b] (%)	Spec[c] (%)	PPV[d] (%)	NPV[e] (%)
Q1. Family history	49.3	87.2	92.4	35.3
Q2. Eczema or red rash	95.9	42.6	84.0	76.9
Q3. Dry skin	67.6	74.5	89.3	42.2
Q4. Itching	90.5	59.6	87.6	66.7
Q5. Occurs within 1 week	87.8	31.9	80.2	45.5
Q6. Occurs within 6 months	90.5	40.4	82.7	57.6
Q7. Previous diagnosis	48.0	100	100	37.9

[a]eCEQ: electronic version of the modified Child Eczema Questionnaire.

[b]NPV: negative predictive value.

[c]PPV: positive predictive value.

[d]Sen: sensitivity.

[e]Spec: specificity.
